# Supplementary material for: Characterization of the Src-regulated kinome identifies SGK1 as a key mediator of Src-induced transformation
Source: Nat Commun. 2019 Jan 17;10:296. doi: 10.1038/s41467-018-08154-1 (PMC6336867; doi:10.1038/s41467-018-08154-1)
Supplement: Supplementary file 1 — Supplementary Information [file 41467_2018_8154_MOESM1_ESM.pdf]

Characterization of the Src-regulated kinome identifies SGK1 as a key mediator of Src-induced transformation

Ma et al.

Supplementary Figures

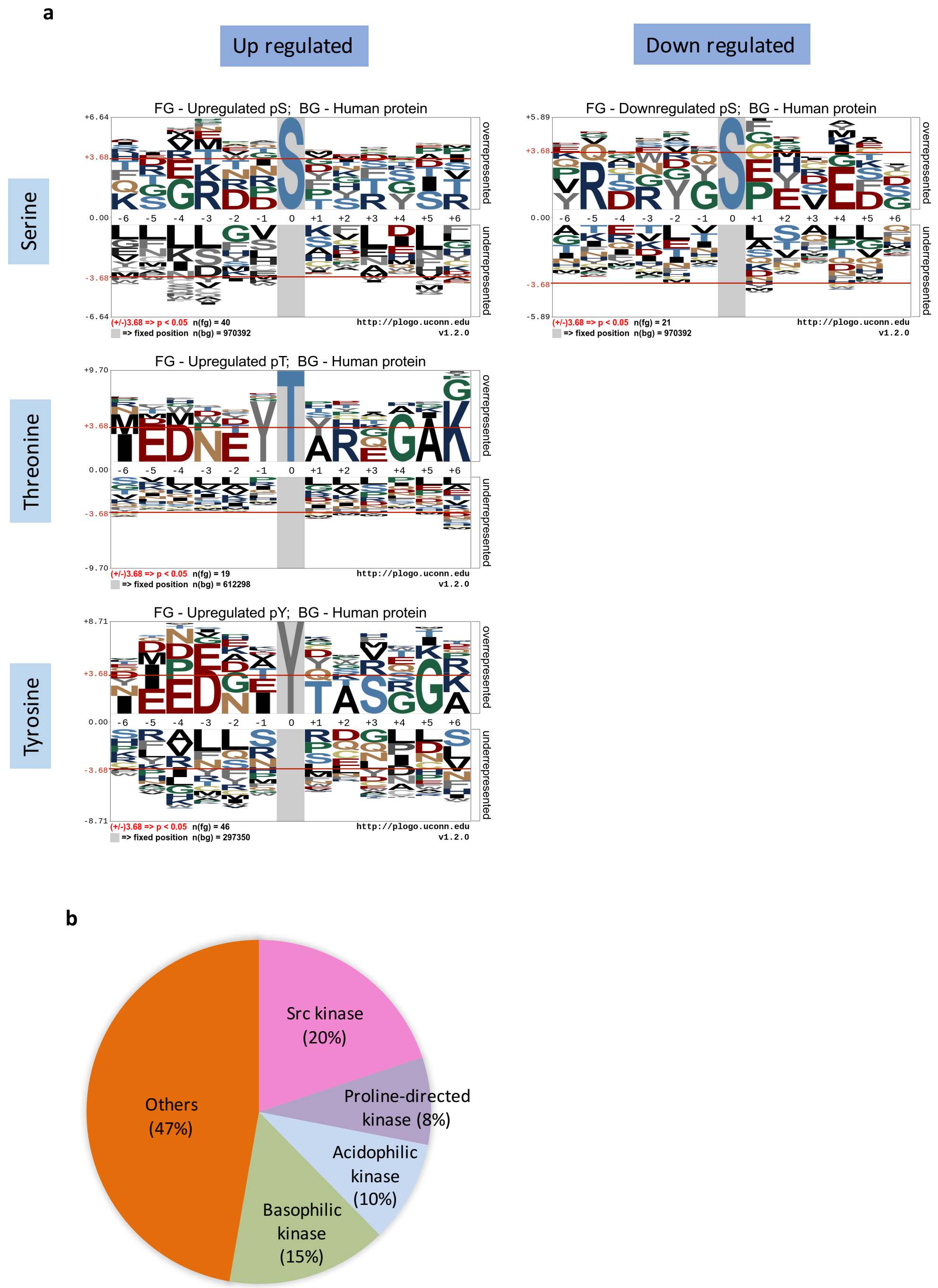

Supplementary Figure 1. Characterization of kinase phosphorylation sites.

(a) Phosphosite sequence enrichment characterized by pLogo. Data shown are for serine (top), threonine (middle) and tyrosine (bottom) sites that are upregulated (left column) and downregulated (right column) in Src-transformed cells.

(b) Contribution of specific sequence motifs to all regulated phosphosites.

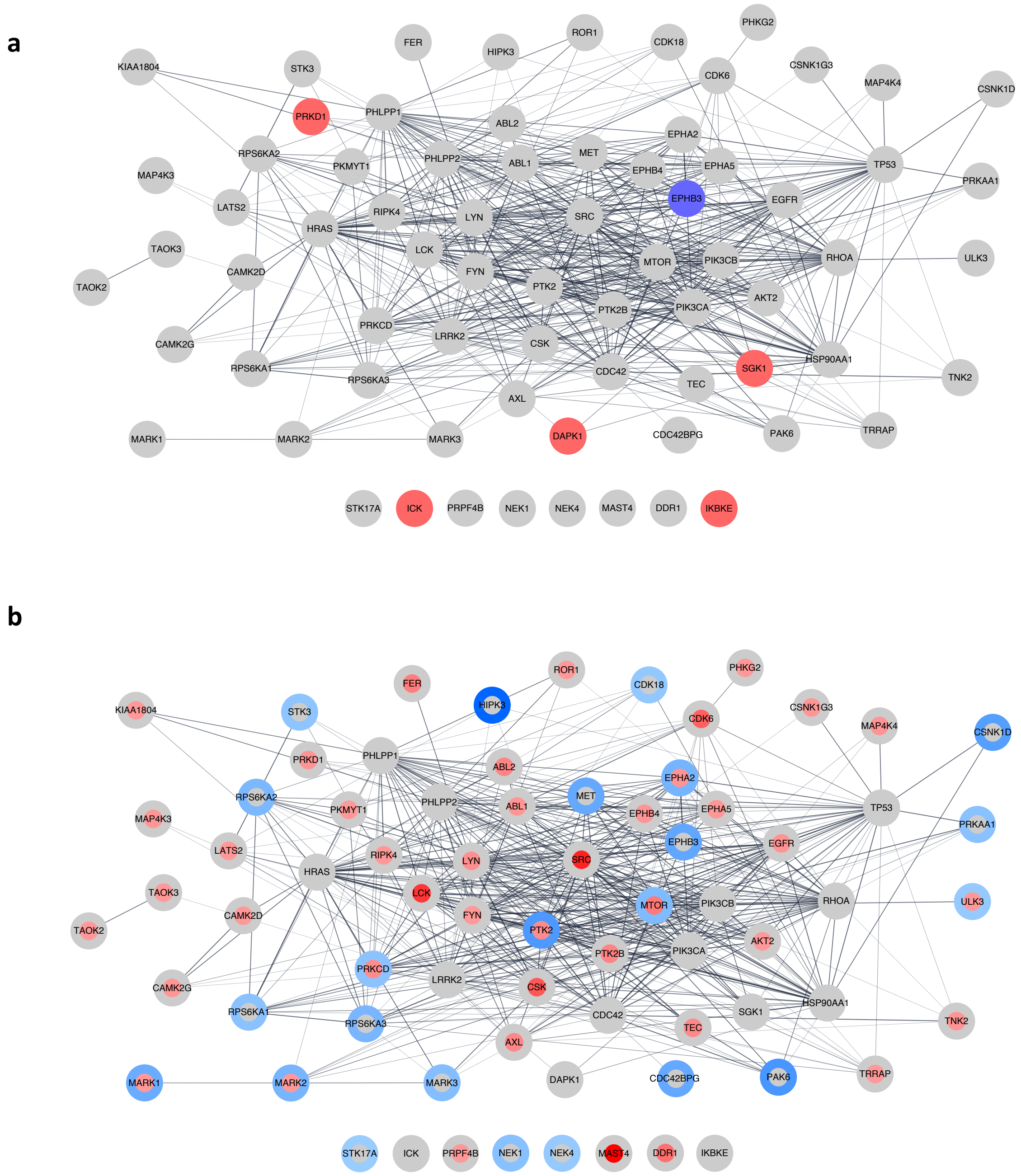

Supplementary Figure 2. Protein-protein interaction networks.

(a-b) Protein-protein interaction networks for kinases exhibiting expression **(a)** or phosphorylation **(b)** changes in Src-transformed cells. Proteins that are up-regulated or down-regulated (Supplementary Figure 2a) or proteins that contain up-regulated or down-regulated phosphosites (Supplementary Figure 2b) are indicated by different colours of the nodes in the interactome. In each panel, warm color indicates up-regulation, while cold color indicates down-regulation. Some kinases exhibit both up- and down-regulated phosphosites and are coloured accordingly. Grey colour only indicates no experimental fold change data is available for those proteins. Fold changes are proportional to the levels of colour darkness.

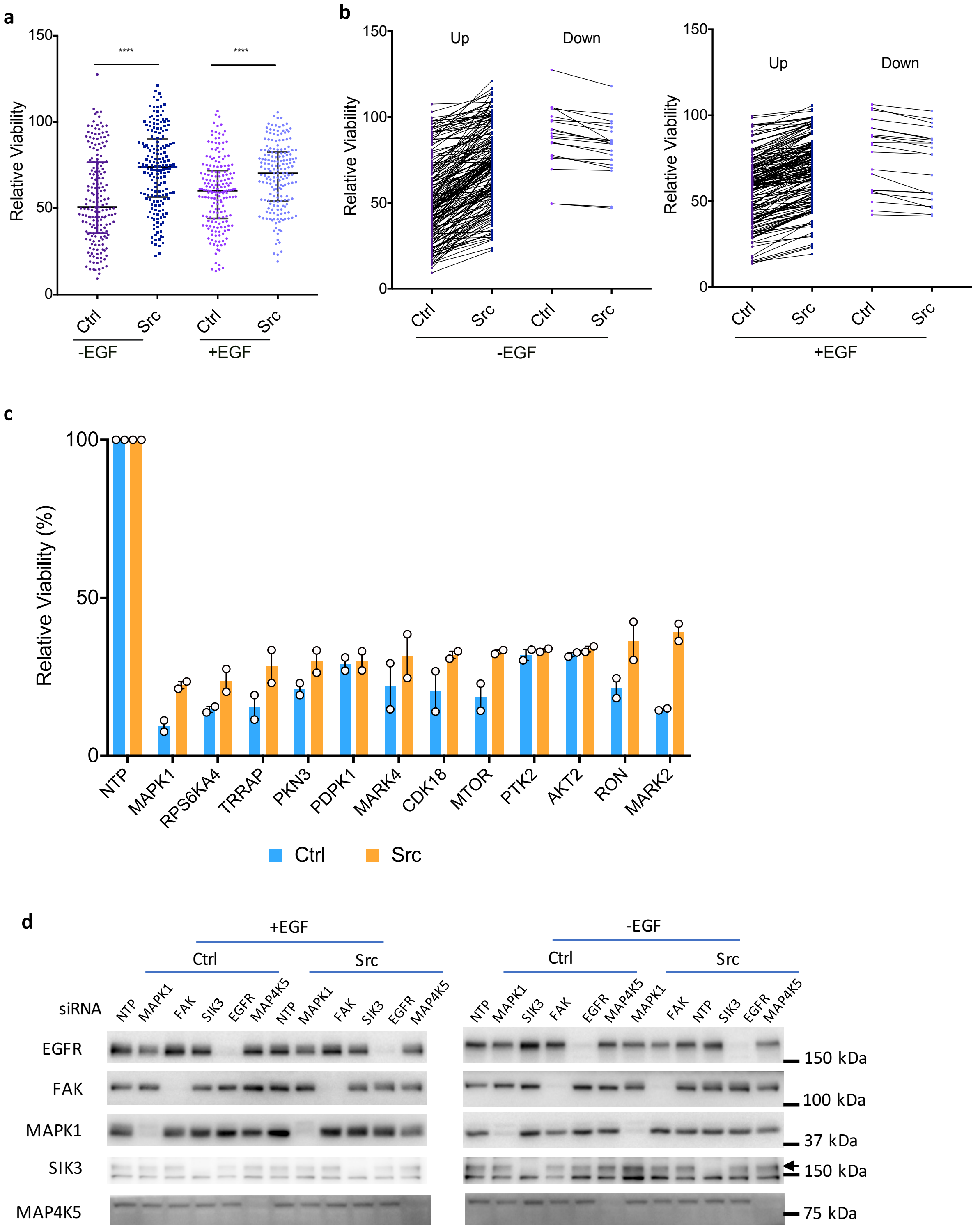

Supplementary Figure 3. Overview of 2D viability screen.

(a-c) MCF-10A_Src cells (Src) are, in general, more resistant to siRNA-mediated knockdown of screen kinases under both +EGF and –EGF conditions than MCF-10A_Ctrl (Ctrl) cells. **(a)** Scatter plots show relative viability following siRNA knockdown compared to mock transfected cells. Targets were robotically reverse transfected in duplicate plates and cell

viability measured at 72 or 96 h post transfection for +EGF and –EGF conditions respectively. **** $p<0.0001$ by ANOVA with Bonferroni's multiple comparisons test.

(b) The relative viability values following knockdown of a given kinase in Ctrl and Src cells are connected to highlight the contrasting effect of knockdown on viability in the two cell types. Up or Down (ratio of relative viability in Src cells to Ctrl cells >1 or <1) are shown separately.

(c) Kinases required for both cell types. Targets identified in Fig. 3c as "essential for both" were plotted. Error bars represent s.e.m. from 2 biological replicates.

(d) Validation of the knockdown of representative targets in 2D screen. Ctrl and Src cells were transfected with the indicated siRNA SMARTpools at 40 nM for 72 h. Cell lysates were Western blotted as indicated. NTP, non-targeting control.

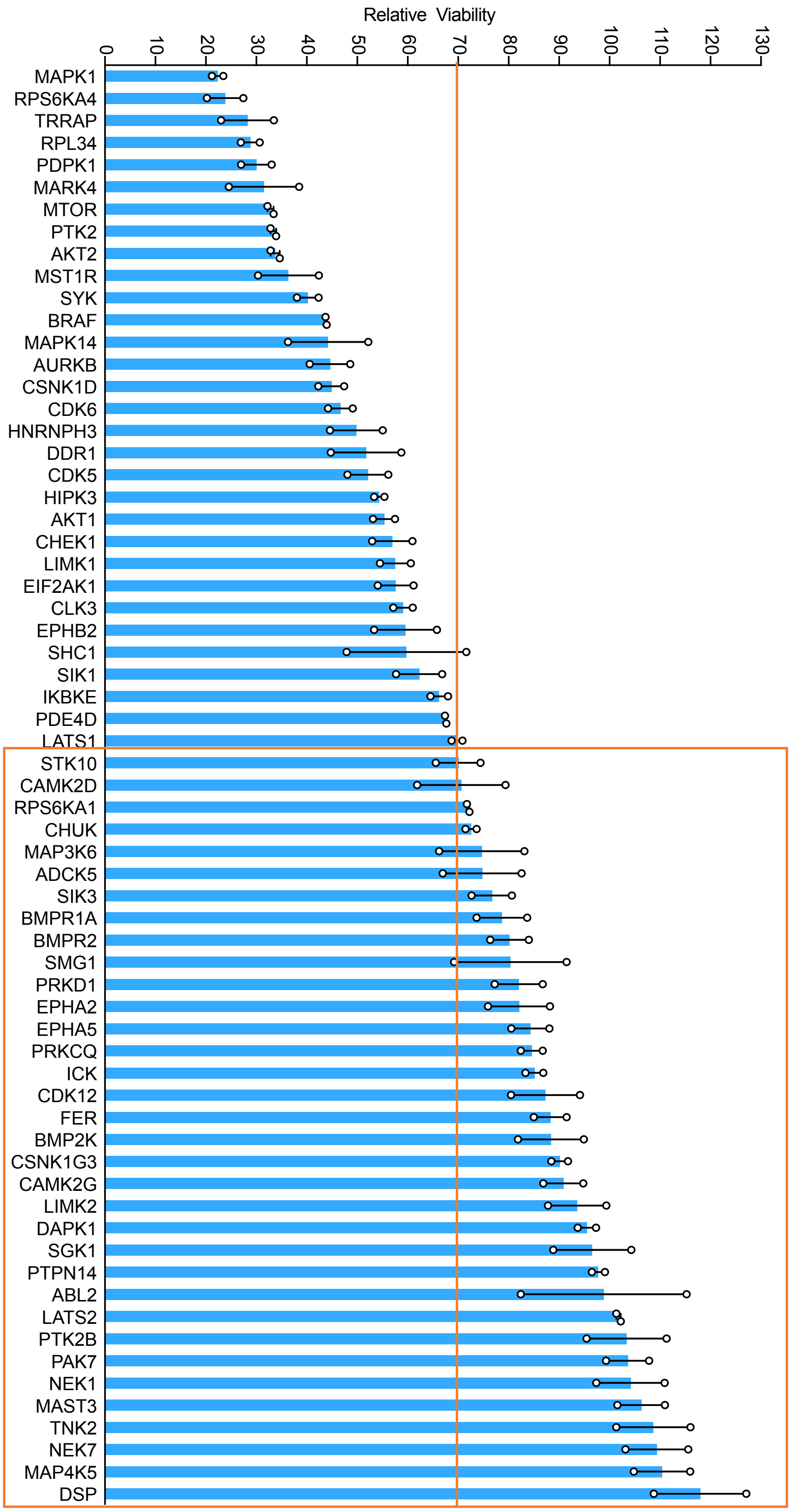

Supplementary Figure 4. Identification of kinases from 2D screen where knockdown affects proliferation < 30%. The data were generated from MCF-10A_Src cells in –EGF 2D culture and are expressed relative to the NTP control which was arbitrarily set at 100%. Error bars represent s.e.m. from 2 biological replicates.

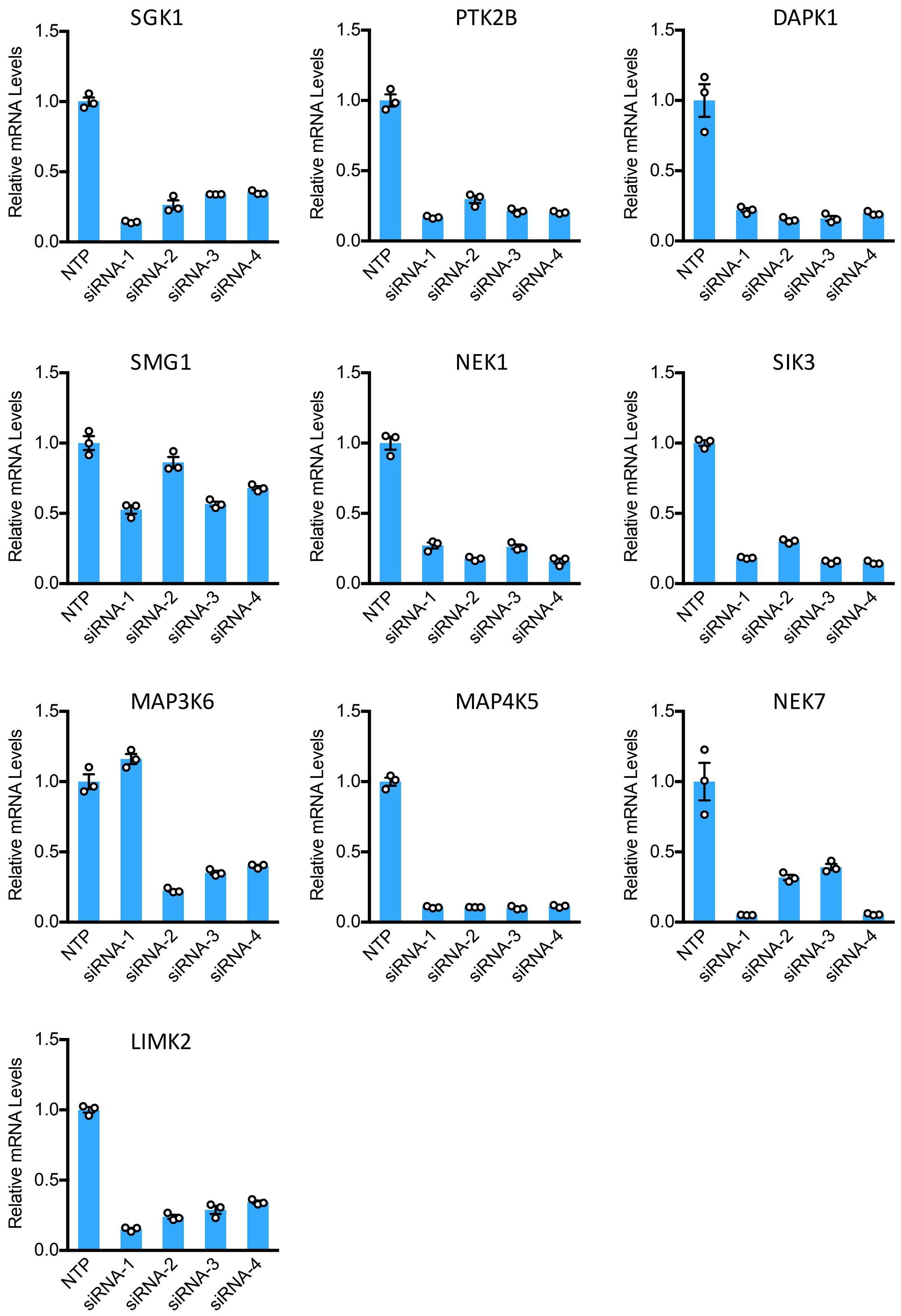

Supplementary Figure 5. Validation of the knockdown of targets in Table 1. MCF-10A_Src cells were transfected with the indicated siRNAs at 40 nM for 48 h. RNA was extracted and subjected to qPCR. Data are expressed relative to the non-targeting control (NTP) which was arbitrarily set at 1. Error bars represent s.d. from 3 replicates.

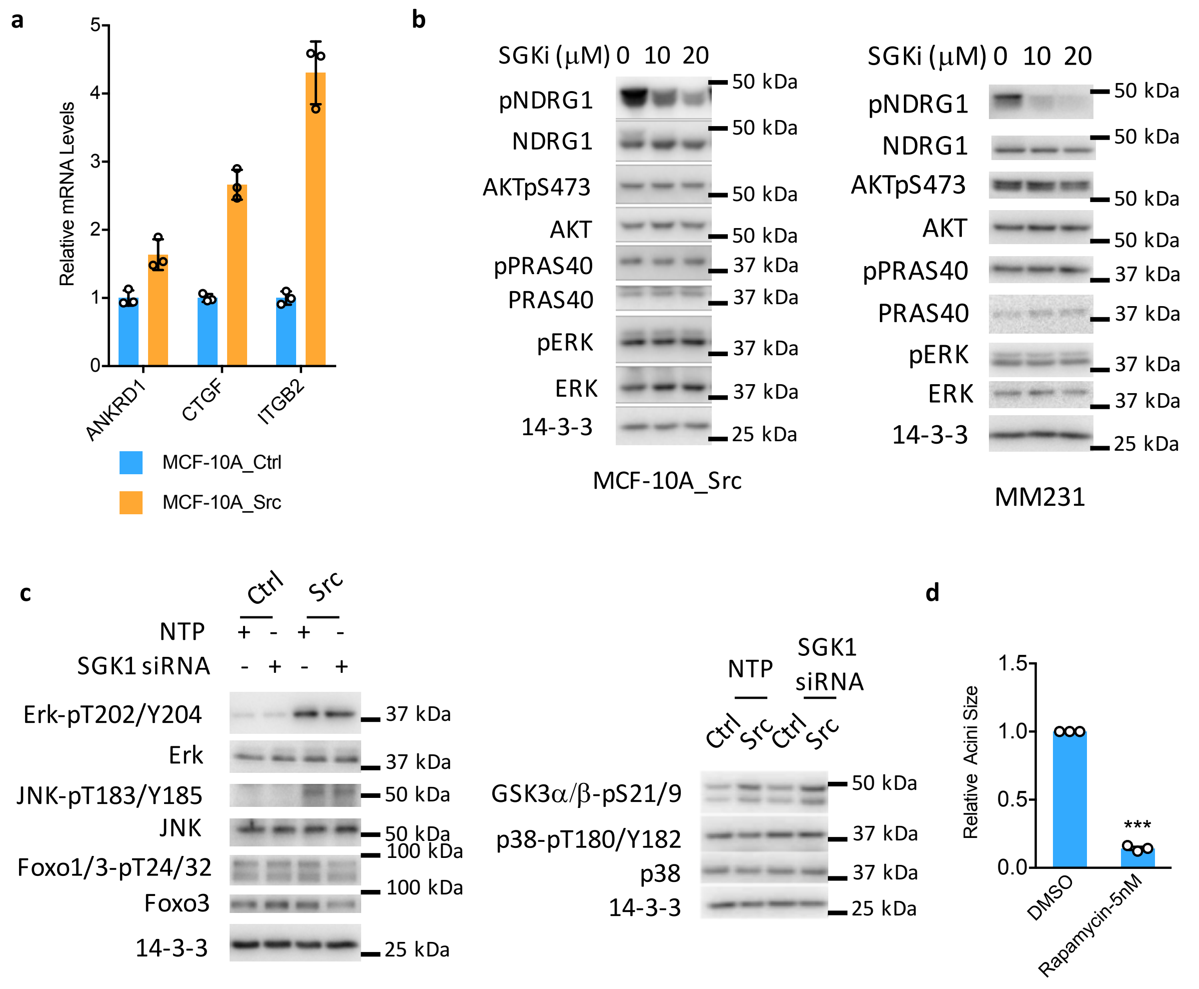

Supplementary Figure 6. Characterization of signalling in Src-transformed MCF-10A cells.

(a) Active Src increases YAP transcriptional activity. qPCR was performed using RNA from MCF-10A_Ctrl and MCF-10A_Src cells cultured in –EGF medium for 24 h before harvesting. Representative results are shown. n=2 biological replicates. Error bars represent s.d. from 3 replicates.

(b) Effect of SGK1 inhibitor on downstream signalling. MCF-10A_Src cells in 3D culture and MDA-MB-231 (MM231) cells in monolayer culture were treated overnight with the SGK1 inhibitor. DMSO was the vehicle control. Cell lysates were Western blotted as indicated. 14-3-3 was the loading control. Representative blots are shown. n=2 biological replicates.

(c) Effect of SGK1 knockdown on downstream signalling. MCF-10A_Ctrl and MCF-10A_Src cells were transfected with SGK1 siRNAs and cultured in –EGF medium for 24 h before harvesting. Cell lysates were Western blotted as indicated. 14-3-3 was the loading control. Representative blots are shown. n=2 biological replicates.

(d) The mTOR inhibitor rapamycin inhibits acini growth. MCF-10A_Src cells were treated with Rapamycin during acini growth. Acini were imaged and quantified from n=3 biological replicates. Error bars represent s.e.m., ***p<0.001 by Student's t-test.

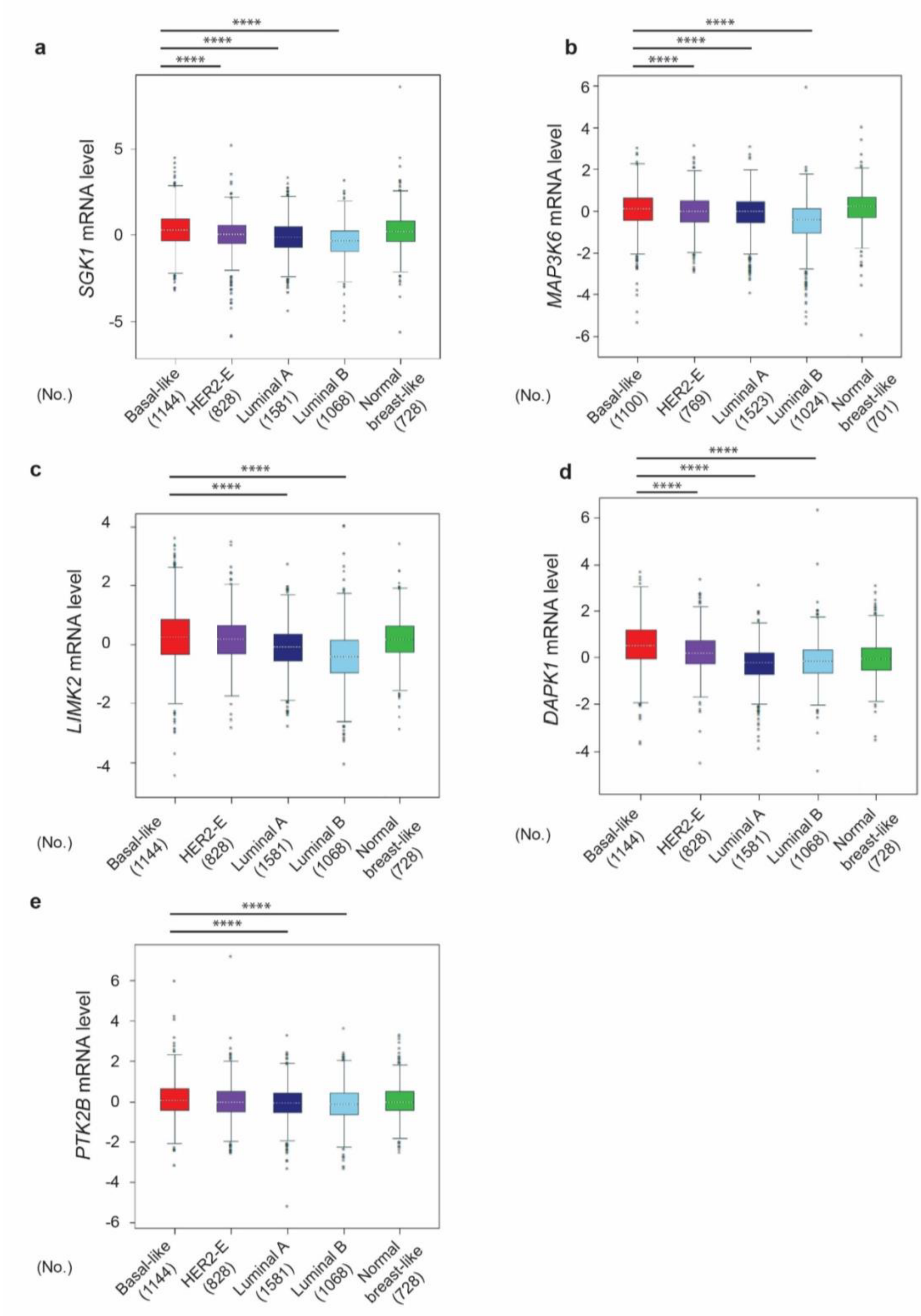

Supplementary Figure 7. Expression of kinase targets across breast cancer subtypes.

(a-e) Expression of validated kinase targets across breast cancer subtypes. Data for SGK1 (a), MAP3K6 (b), LIMK2 (c), DAPK1 (d) and PTK2B/FAK2 (e) were extracted from Breast Cancer Gene-Expression Miner 4.0. mRNA levels were plotted on a box and whisker graph, with the box spanning the lower quartile (Q1) to the upper quartile (Q3), with a horizontal line

marking the median. At the bottom and the top of the box, whiskers indicate the distance between the Q1, respectively Q3, and 1.5 times the interquartile range, that is: Q1-1.5*(Q3-Q1) and Q3+1.5*(Q3-Q1). Finally, stars indicate outliers, if there is any, that is, patients with values below or above the end of the whiskers. ****p<0.0001 by Welch's t-test, the default statistical analysis with this tool.

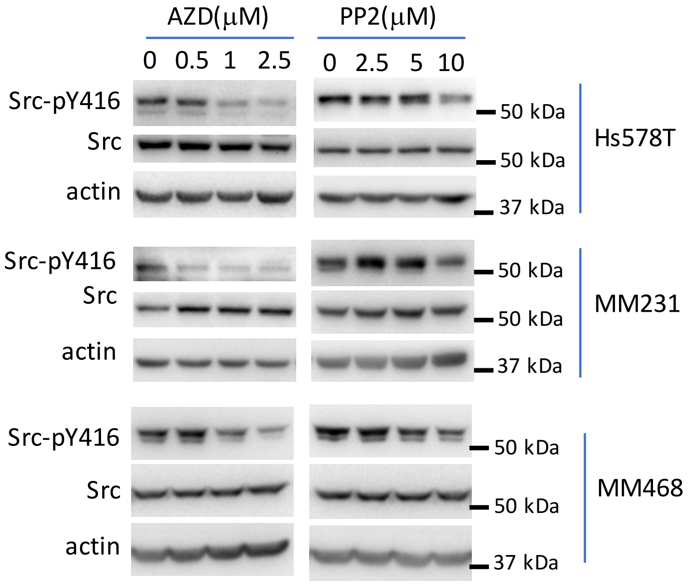

Supplementary Figure 8. Effect of Src inhibitor on Src activity.

Cell lysates used in Figure 7b and 7c were Western blotted as indicated. Actin was the loading control.

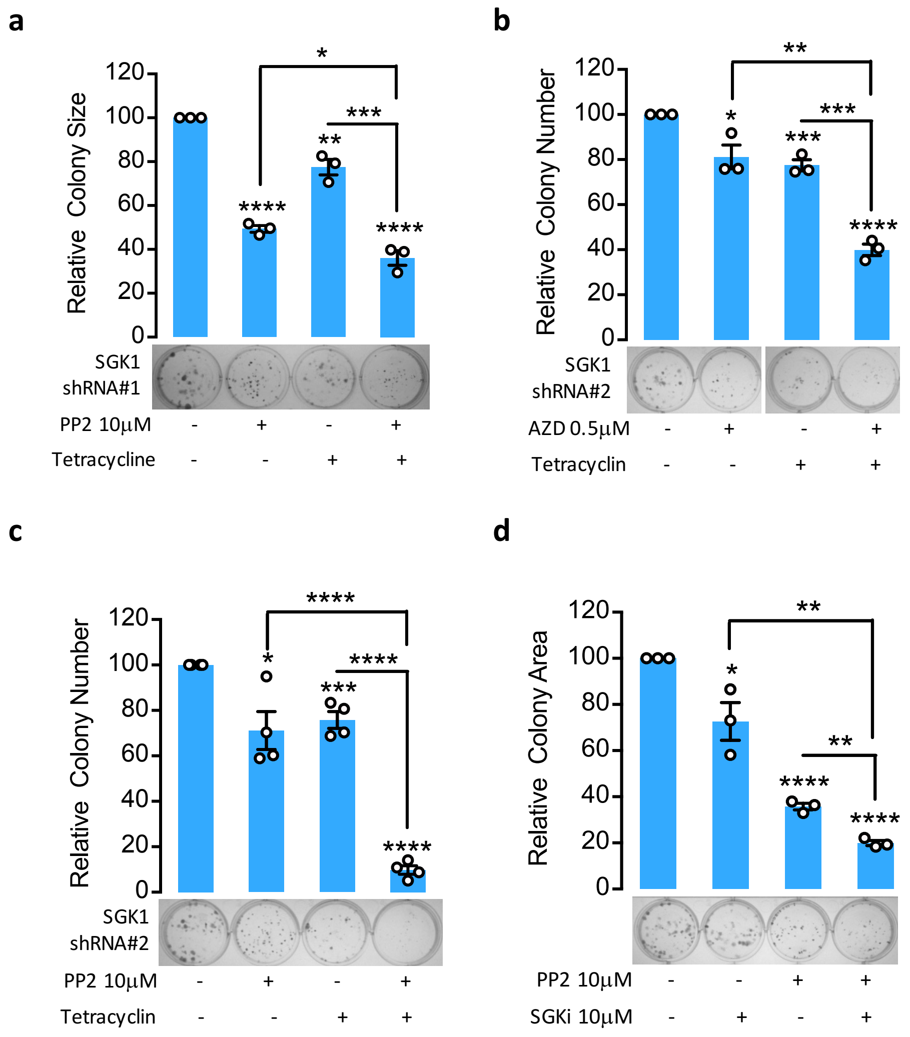

Supplementary Figure 9. Effect of combined SGK1 and Src knockdown/inhibition in MDA-MB-231 TNBC cells.

(a-d) Effect of combining SGK1 knockdown or SGKi with Src inhibitor (AZD or PP2) on colony formation. Shown are data for colony formation assays using MDA-MB-231 (MM231) cells stably transfected with tetracyclin inducible SGK1 shRNA#1 treated with tetracyclin +/- PP2 **(a)**, tetracyclin-inducible shRNA#2 treated with tetracyclin +/- AZD **(b)**, tetracyclin-inducible shRNA#2 treated with tetracyclin +/- PP2 **(c)**, or MDA-MB-231 cells treated with SGKi +/- PP2 **(d)**. DMSO was the vehicle control. Data are expressed relative to the DMSO control which was arbitrarily set at 100. Results were quantified from n=3 (a, b and d) or n=4 (c) biological replicates. For **(d)**, the coefficient of drug interaction (CDI) was 0.77, indicating modest synergy. Error bars represent s.e.m., $^*p<0.05$, $^{**}p<0.01$, $^{***}p<0.001$, $^{****}p<0.0001$ by Student's t-test.

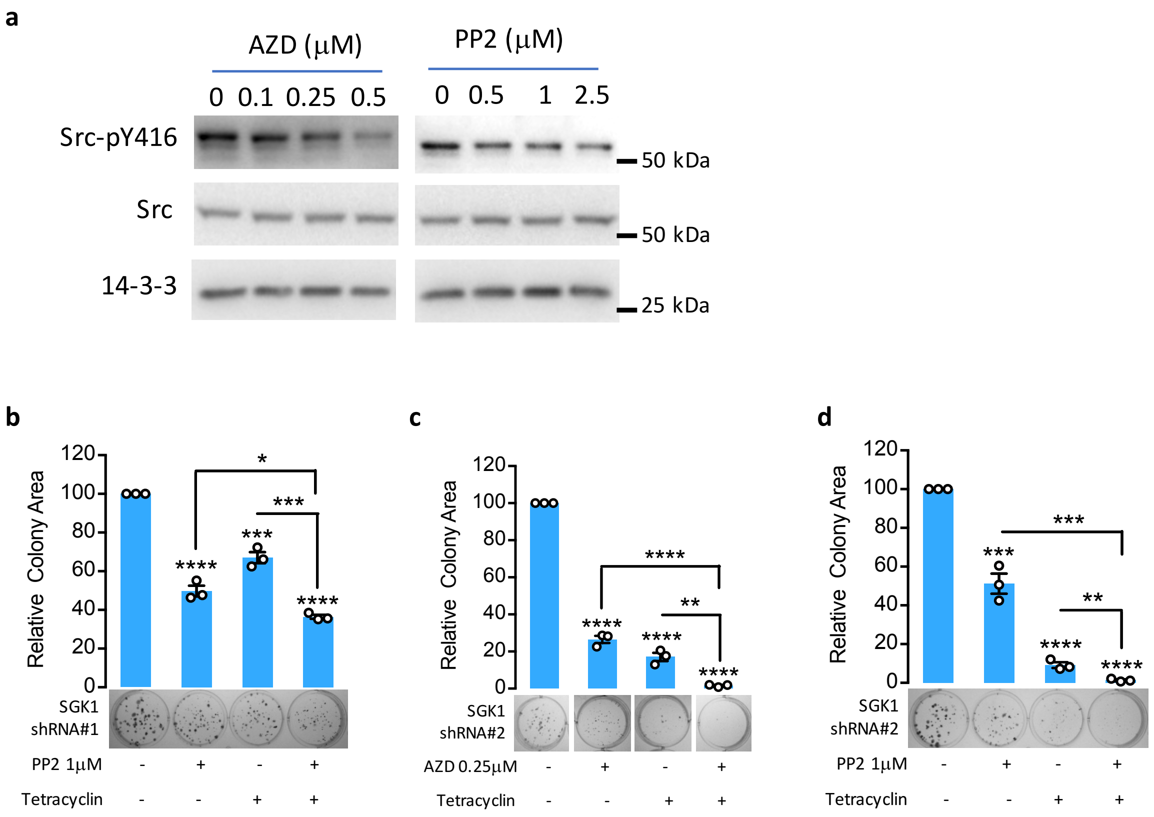

Supplementary Figure 10. Combined effect of SGK1 knockdown and Src inhibition in A549 NSCLC cells.

(a) Effect of Src inhibitor on Src activity. Lysates from A549 cells treated for 1 h with AZD0530 (AZD) or PP2 were Western blotted as indicated. Representative blots are shown from n=2 biological replicates.

(b-d) Colony formation assays using A549 cells stably transfected with tetracyclin inducible SGK1 shRNA#1 treated with tetracyclin +/- PP2 **(b)**, tetracyclin-inducible shRNA#2 treated with tetracyclin +/- AZD **(c)** or tetracyclin-inducible shRNA#2 treated with tetracyclin +/- PP2 **(d)**. DMSO was the vehicle control. Data are expressed relative to the DMSO control which was arbitrarily set at 100. Results were quantified from n=3 biological replicates. Error bars represent s.e.m., *p<0.05, **p<0.01, ***p<0.001, ****p<0.0001 by Student's t-test.

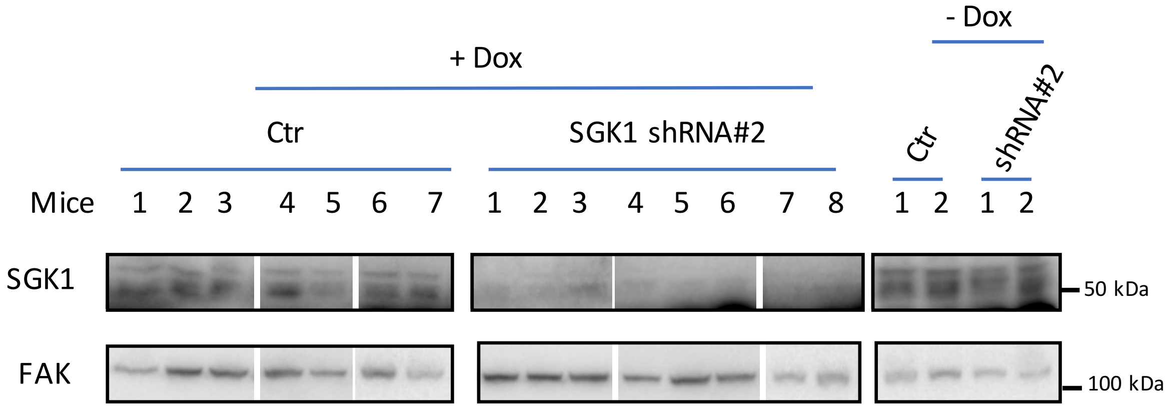

Supplementary Figure 11. SGK1 expression levels in tumor xenografts.

Tumor xenografts from mice used to generate Fig. 9b (+Dox), as well as tumor xenografts (2 mice from each group) from mice without doxycycline treatment (-Dox) were collected. Tumor xenograft lysates were Western blotted as indicated. FAK was used as a loading control.

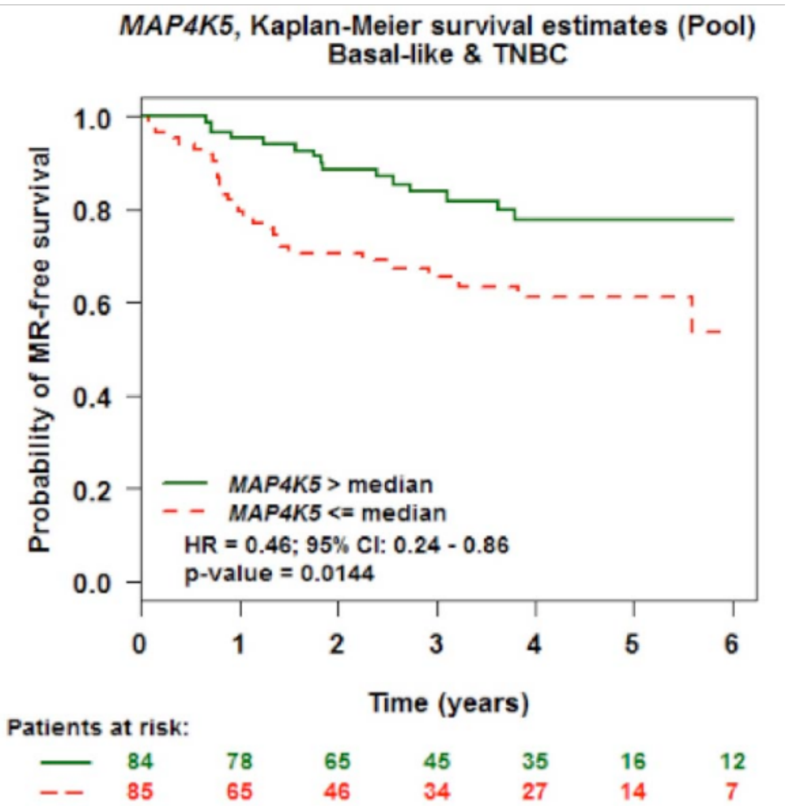

Supplementary Figure 12. Expression of MAP4K5 and survival estimates in TNBC patients. Kaplan-Meier survival estimates of TNBC patients with high or low expression of MAP4K5. Data were extracted from Breast Cancer Gene-Expression Miner 4.0.

Fig. 4c

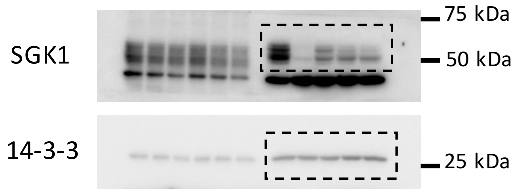

Fig. 5b

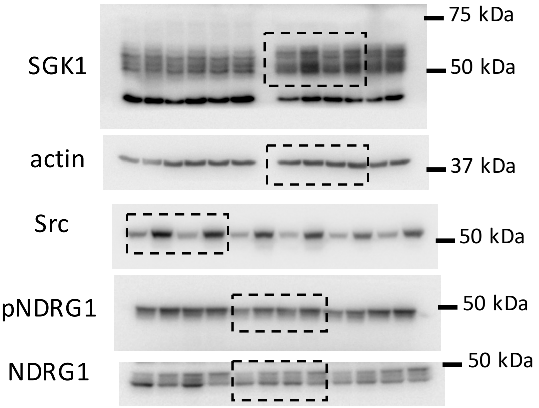

Fig. 5c

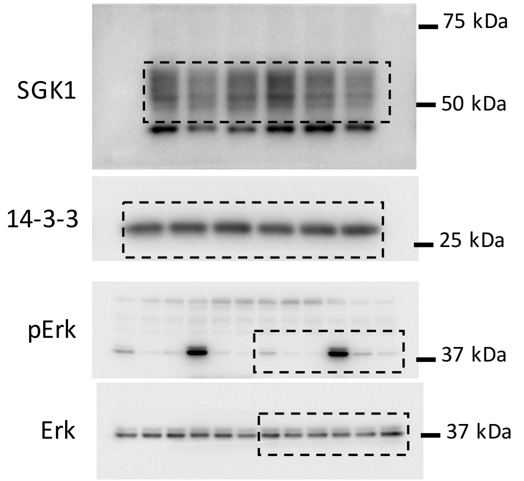

Fig. 5d

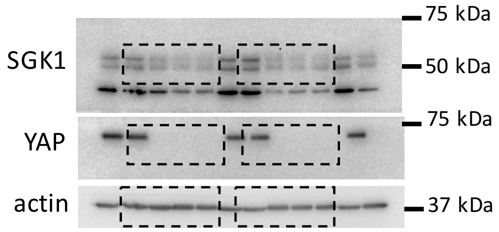

Fig. 5e

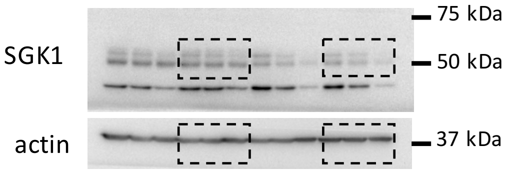

Fig. 5f

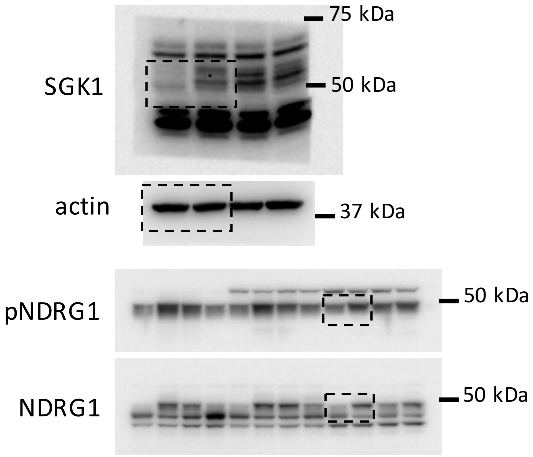

Fig. 6c

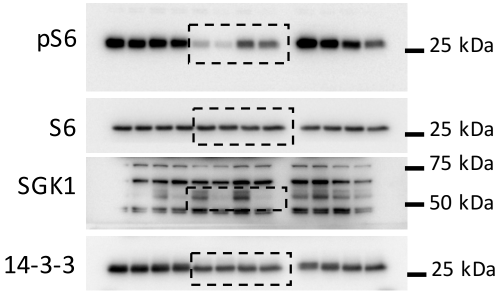

Fig. 6d

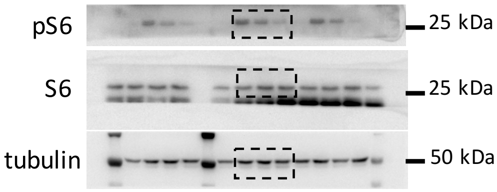

Fig. 7a

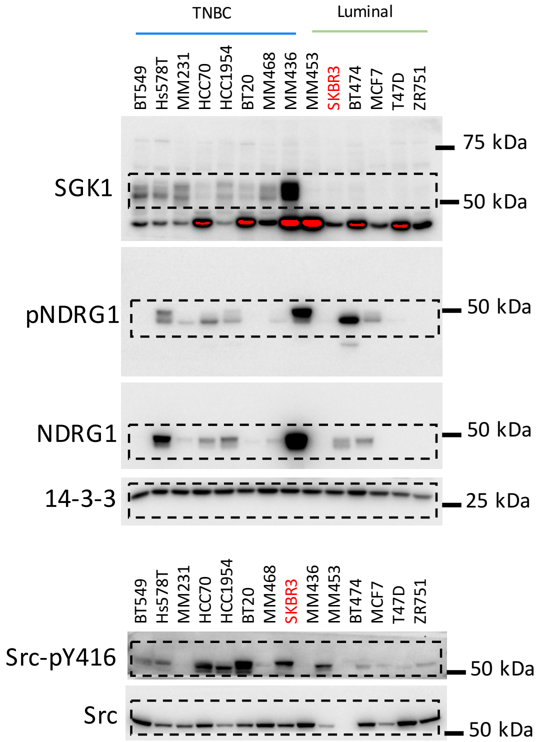

Fig. 7b

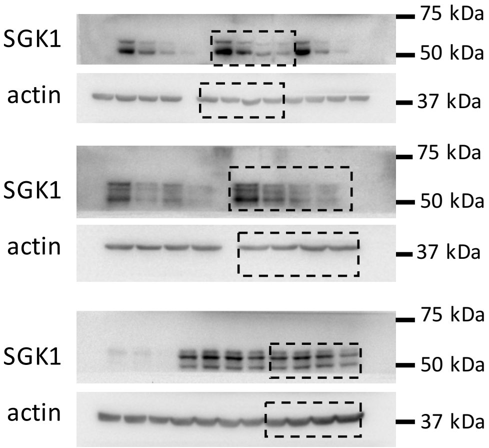

Fig. 7c

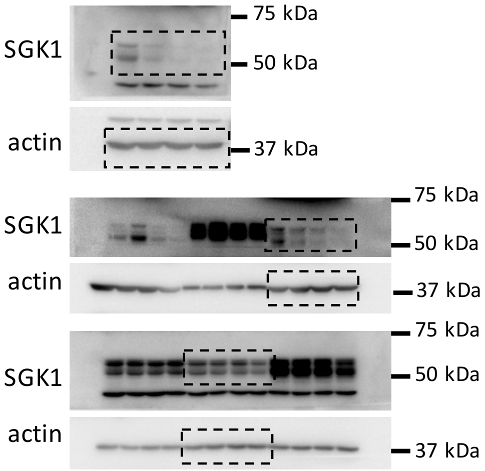

Fig. 7d

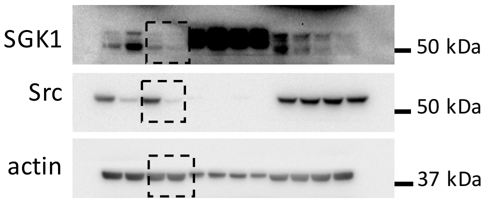

Fig. 7e

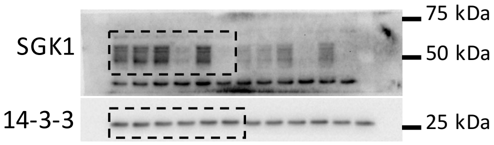

Fig. 8a

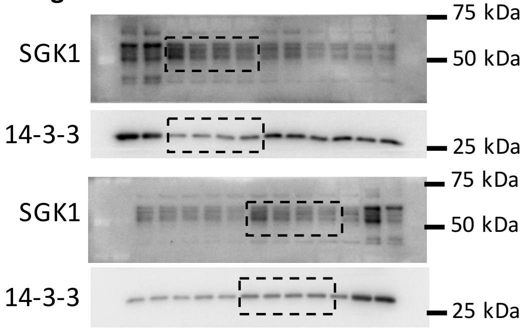

Fig. 8b

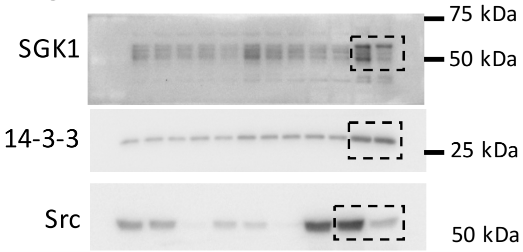

Fig. 8c

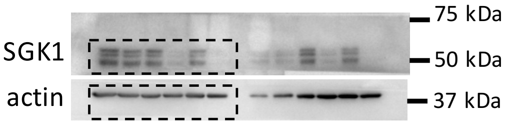

Supplementary Figure 13. Fully uncropped scans of all blots presented in the main figures. Lanes used in figures are lined out by dashed boxes and the antibodies used for detection are indicated. To allow concomitant detection of more than one protein on the same gel, polyvinylidene difluoride (PVDF) membranes have been cut with a knife at the specified molecular weights.

Supplementary Tables

	Uniprot_AC	UniProt name	Protein name used in Figures	Average fold change
**UP**	O00141-2	SGK1	SGK1	4.02
	P53355	DAPK1	DAPK1	2.29
	Q14164	IKKE	IKKE	2.06
	Q9UPZ9	ICK	ICK	1.58
	Q15139	KPCD1	PKD1	1.51
**DOWN**	P54753	EPHB3	EPHB3	0.65

Supplementary Table 1. List of kinases exhibiting expression changes of ≥1.5 fold in either direction.

MS-based kinomic profiling results generated from MCF-10A_Ctrl and MCF-10A_Src cells as described in Methods. 6 kinases exhibiting ≥1.5 fold expression changes were identified. There were 5 up-regulated kinases and 1 down-regulated kinase.

Up-regulated at expression level
Down-regulated at expression level
Changed at both phosphorylation and expression level

Accession number	UniProt name	Protein name used in Figures	pSite position	Fold change in MCF-10A_Src	Fold change in MM231 (DMSO/AZD)
P12931-2	SRC	SRC	Y425	25.146	18.53
P42684	ABL2	ABL2	Y439	6.254	>10
P42680	TEC	TEC	Y519	5.358	>10
Q05397-2	FAK1	FAK	Y688	4.781	>10
Q05655	KPCD	KPCD	Y334	4.522	>10
Q05655	KPCD	KPCD	Y313	4.141	11.59
Q05397	FAK1	FAK	Y861	3.064	>10
P07948	LYN	LYN	Y397	2.975	80.65
Q07912-3	ACK1	TNK2	Y905	2.639	>10
P29317	EPHA2	EPHA2	Y575	2.623	0.85
P42684	ABL2	ABL2	S602	2.591	5.73
P29317	EPHA2	EPHA2	Y772	2.538	2.63
Q7KZI7	MARK2	MARK2	S479	2.254	>10
P29317	EPHA2	EPHA2	T774	2.220	>10
Q13555-8	KCC2G	CAMK2G	T287	2.117	1.53
Q8IVH8	M4K3	MAP4K3	S329	1.885	>10
Q9NRM7	LATS2	LATS2	T279	1.841	0.65
P06241	FYN	FYN	S21	1.837	2.31
P42684	ABL2	ABL2	Y718	1.836	>10
P15735	PHKG2	PHKG2	S324	1.749	0.64
Q05397	FAK1	FAK	Y576	1.606	3.01
Q13557	KCC2D	CAMK2D	S280	1.523	1.10

Supplementary Table 2. List of overlapping sites identified by small scale kinome profiling of MDA-MB-231 TNBC cells.

MDA-MB-231 (MM231) cells were treated with AZD or DMSO vehicle control for 16 h. Cell lysates were collected and subjected to MS-based kinomic profiling as described in Methods. The table indicates sites that exhibited increased phosphorylation in MCF10A_Src cells and that were detected in the MDA-MB-231 dataset. Column "Fold change in MM231 (DMSO/AZD)" gives the DMSO/AZD ratio for these sites. Phosphorylation has been normalized for total kinase protein. Those shaded in red (defined as Match, 18/22=81.82%) are increased in MCF-10A_Src cells and decreased by AZD treatment of MDA-MB-231 cells. Those shaded in blue (defined as Opposing, 2/22=9.09%) exhibit an opposing change and those in beige (defined as Neutral, 2/22=9.09%) are increased in MCF-10A_Src cells but do not change (<1.5 fold change) upon AZD treatment of MDA-MB-231s. The use of > indicates that

the phosphosite was not detected in the AZD-treated sample and so the value has been obtained via imputation.

Match	18/22=81.82%
Opposing	2/22=9.09%
Neutral	2/22=9.09%

Kinase	siRNA	Fold Change (% of NTP)	Effective siRNAs
SGK1	SGK1 siRNA1	49.6	4/4
	SGK1 siRNA2	62.9	
	SGK1 siRNA3	66.6	
	SGK1 siRNA4	65.2	
MAP4K5	MAP4K5 siRNA1	146.9	4/4
	MAP4K5 siRNA2	178.6	
	MAP4K5 siRNA3	169.0	
	MAP4K5 siRNA4	154.0	
NEK1	NEK1 siRNA1	34.6	4/4
	NEK1 siRNA2	46.4	
	NEK1 siRNA3	48.1	
	NEK1 siRNA4	54.9	
NEK7	NEK7 siRNA1	43.7	4/4
	NEK7 siRNA2	36.0	
	NEK7 siRNA3	29.8	
	NEK7 siRNA4	33.0	
DAPK1	DAPK1 siRNA1	67.0	4/4
	DAPK1 siRNA2	56.4	
	DAPK1 siRNA3	58.4	
	DAPK1 siRNA4	37.2	
LIMK2	LIMK2 siRNA1	63.5	3/4
	LIMK2 siRNA2	69.1	
	LIMK2 siRNA3	86.7	
	LIMK2 siRNA4	107.9	
PTK2B	PTK2B siRNA1	116.7	3/4
	PTK2B siRNA2	62.9	
	PTK2B siRNA3	79.5	
	PTK2B siRNA4	71.0	
MAP3K6	MAP3K6 siRNA1	111.9	2/4
	MAP3K6 siRNA2	62.8	
	MAP3K6 siRNA3	56.2	
	MAP3K6 siRNA4	104.3	
SMG1	SMG1 siRNA1	68.3	4/4
	SMG1 siRNA2	73.2	
	SMG1 siRNA3	42.3	
	SMG1 siRNA4	48.6	
SIK3	SIK3 siRNA1	111.2	3/4
	SIK3 siRNA2	78.3	
	SIK3 siRNA3	69.6	
	SIK3 siRNA4	61.8	

Supplementary Table 3. Deconvolution data for validated targets from the 3D screen.

See also Fig. 4D. The numbers in column "Fold Change (% of NTP)" indicate acini size expressed relative to the value for the non-targeting control (NTP), which was arbitrarily set at 100.

Primers for Quantitative real-time PCR

Name	Primer (5' to 3')
GAPDH-L1	AGCCACATCGCTCAGACAC
GAPDH-R1	AATACGACCAAATCCGTTGACT
ANKRD1-L1	GATCGAATTCCGTGATATGCT
ANKRD1-R1	AAACATCCAGGTTTCCTCCA
CTGF-L1	CCTGCAGGCTAGAGAAGCAG
CTGF-R1	TGGAGATTTTGGGAGTACGG
SGK1-L1	GACAGGACTGTGGACTGGTG
SGK1-R1	TTTCAGCTGTGTTTCGGCTA
DAPK1-L1	CCCTTGTCCCAGTTGAAGAA
DAPK1-R1	CCGGTCGAGGAACATTCA
PTK2B(FAK2)-L1	ATGGGAGAAACCAGAGATGC
PTK2B(FAK2)-R1	TCTGTCTTCCAGGGCTATTCA
SMG1-L1	CCACAGTAGAGCGTTGTCAAGA
SMG1-R1	GGCAGTGATGAACTGACACAC
NEK1-L1	AAAAAGTGTGGGAAGAGCATTT
NEK1-R1	TGGAGAGCCACCTGTTTCAT
SIK3-L1	TGGACGAGGAGAGCTCAGA
SIK3-R1	GCAGAGTGTTGGAGTCCTTGT
MAP3K6-L2	TCACACCTTGCACTGAGACC
MAP3K6-R2	GTCAGCTGCTTTCCCATACC
MAP4K5-L1	TGAACGGACAGCTTCAGAAA
MAP4K5-R1	CCCATTTCATCTCGTGCTTC
NEK7-L1	CATCTGGTCTCTTGGCTGTCT
NEK7-R1	TCATTTTGTCACCATAGAAAGGAC
LIMK2-L1	GAATCTGGAGGGGACACTGA
LIMK2-R1	GCTGATGTCACGGCTGAAC

siRNA sequences (Used in Figure 5D)

	**Catalog Number**	**Sequence**
YAP siRNA #2	J-012200-06	UGAGAACAAUGACGACCAA
YAP siRNA #3	J-012200-07	GGUCAGAGAUACUUCUUAA
YAP siRNA #4	J-012200-08	CCACCAAGCUAGAUAAAGA

SGK1 shRNAs sequences (5' to 3') (Used in Figure 7E, 7F, 8C, 8D and Figure 9)

SGK1 shRNA #1 sense	CCGGCGGAATGTTCTGTTGAAGAATCTCGAGATTCTTCAACAG AACATTCCGTTTTTG
SGK1 shRNA #1 antisense	AATTCAAAAACGGAATGTTCTGTTGAAGAATCTCGAGATTCTT CAACAGAACATTCCG

SGK1 shRNA #2 sense	CCGGCTGGAAGCTTAGCAATCTTATCTCGAGATAAGATTGCTAAGCTTCCAGTTTTTG
SGK1 shRNA #2 antisense	AATTCAAAAACTGGAAGCTTAGCAATCTTATCTCGAGATAAGATTGCTAAGCTTCCAG

Supplementary Table 4. List of primers for PCR, siRNA sequences and SGK1 shRNA sequences.

Gene Symbol	GENE ID	Duplex Catalog Number	Label in paper	Sequence
SGK1	6446	D-003027-09	siRNA-1	GGAGCUGUCUUGUAUGAGA
SGK1	6446	D-003027-10	siRNA-2	UGAACAUCGUUUAUAGAGA
SGK1	6446	D-003027-11	siRNA-3	CAGCUGAAACCAAAUAUUA
SGK1	6446	D-003027-12	siRNA-4	UCAUGGAGAUUAAGAGUCA
MAP3K6	9064	D-003969-01	siRNA-1	UGGAGAAGAUGCAGUAUUA
MAP3K6	9064	D-003969-02	siRNA-2	CCAAAGAGCUCCGGCUAAU
MAP3K6	9064	D-003969-03	siRNA-3	GGACUACUCGGCCAUCAUU
MAP3K6	9064	D-003969-04	siRNA-4	CCGAAGCGCUGCCUCAGUU
MAP4K5	11183	D-003589-01	siRNA-1	GUAAGAAACUGGCCUCUGA
MAP4K5	11183	D-003589-06	siRNA-2	GCGGAUCACUUCAAGAUAU
MAP4K5	11183	D-003589-07	siRNA-3	GCAUAGAGAUAUCAAAGGU
MAP4K5	11183	D-003589-08	siRNA-4	CGAAUCAGGUAGUUCAGUU
NEK1	4750	D-004864-01	siRNA-1	GAAAUAAACCCAUCAGCUA
NEK1	4750	D-004864-02	siRNA-2	GAGAGAAGUUGCAGUAUUG
NEK1	4750	D-004864-03	siRNA-3	GAAUUUGGCUUAUCAGAAU
NEK1	4750	D-004864-04	siRNA-4	GGGAAGCUAUGCAGAAUAA
NEK7	140609	D-003795-07	siRNA-1	GAUACAACUUCAAAUCUGA
NEK7	140609	D-003795-08	siRNA-2	GAUAUGGGCUAUAAUACAU
NEK7	140609	D-003795-09	siRNA-3	GAAAAUUGGUCGCGGACAA
NEK7	140609	D-003795-10	siRNA-4	AGACGUCACCUAUGUUUAU
LIMK2	3985	D-003311-06	siRNA-1	GAUCUGAACUCGCACAACU
LIMK2	3985	D-003311-07	siRNA-2	UGACUGAGGUGAAAGUGAU
LIMK2	3985	D-003311-08	siRNA-3	GACAUCAGCCGCUCAGAAU
LIMK2	3985	D-003311-09	siRNA-4	CUAAGGCGCAGUAACAGUA
PTK2B	2185	D-003165-05	siRNA-1	GAACAUGGCUGACCUCAUA
PTK2B	2185	D-003165-06	siRNA-2	GGACCACGCUGCUCUAUUU
PTK2B	2185	D-003165-07	siRNA-3	GGACGAGGACUAUUACAAA
PTK2B	2185	D-003165-13	siRNA-4	GAGGAAUGCUCGCUACCGA
DAPK1	1612	D-004417-02	siRNA-1	GAAGAAAUGCCGUGAGAAA
DAPK1	1612	D-004417-04	siRNA-2	GAACAGGUUUGGAAAUGAU
DAPK1	1612	D-004417-07	siRNA-3	GAUCAAGCCUAAAGAUACA
DAPK1	1612	D-004417-21	siRNA-4	GGAAACAAUCCGUUCGCUU
SMG1	23049	D-005033-01	siRNA-1	GUGAAGAUGUUCCCUAUGA
SMG1	23049	D-005033-02	siRNA-2	GAGGUUAGCUGCGGAAAGA
SMG1	23049	D-005033-03	siRNA-3	GGUCAGACAUCCACCAGAA
SMG1	23049	D-005033-04	siRNA-4	UAACUUGGCUCAGCUGUAU
SIK3	23387	D-004779-01	siRNA-1	GCGCCAGGCUUUAUCUUAU
SIK3	23387	D-004779-05	siRNA-2	GAACAGCGACGAUGCUUAU
SIK3	23387	D-004779-06	siRNA-3	GCACUAACCUGCUUGGGUA
SIK3	23387	D-004779-20	siRNA-4	GGAGCAGGCAGGCGUGUAA

Supplementary Table 5. List of siRNA information for deconvolution validation.
