# Supplementary material for: Characterization of the Src-regulated kinome identifies SGK1 as a key mediator of Src-induced transformation
Source: Nat Commun. 2019 Jan 17;10:296. doi: 10.1038/s41467-018-08154-1 (PMC6336867; doi:10.1038/s41467-018-08154-1)
Supplement: Supplementary file 3 — Description of Additional Supplementary Files [file 41467_2018_8154_MOESM3_ESM.docx]

**Description of Additional Supplementary Files**

**File Name:** Supplementary Data 1

**Description:** List of kinase phosphorylation sites exhibiting ≥1.5 fold change in either direction. MS-based kinomic profiling results generated from MCF-10A_Ctrl and MCF-10A_Src cells as described in Methods. 135 kinase phosphorylation sites exhibiting ≥1.5 fold change in either direction were identified. The 135 phosphorylated sites correspond to a total of 59 kinases. There were 106 up-regulated sites and 29 down-regulated sites.

**File Name:** Supplementary Data 2

**Description:** Compiled list of kinases exhibiting phosphorylation and/or expression changes of ≥1.5 fold in either direction. List of all kinases used to generate Supplementary Data 1 and Supplementary Table 1.

**File Name:** Supplementary Data 3

**Description:**  List of targets for siRNA screen.

**File Name:** Supplementary Data 4

**Description**: Impact of kinase knockdown on cell viability under 2D (monolayer) conditions. List of 2D viability results used to generate Figure 3 and Supplementary Figure 3.

**File Name:** Supplementary Data 5

**Description:** Comparison of target dependencies under 2D and 3D growth conditions. The numbers in column B indicate relative acini size of MCF-10A_Src cells transfected with different siRNA SMARTpools as indicated in column A, expressed relative to the value of non-targeting control, which was arbitrarily set at 100. The numbers in column C and D are 2D viability results extracted from Supplementary Data 4.
