# Supplementary material for: Characterization of the Src-regulated kinome identifies SGK1 as a key mediator of Src-induced transformation
Source: Nat Commun. 2019 Jan 17;10:296. doi: 10.1038/s41467-018-08154-1 (PMC6336867; doi:10.1038/s41467-018-08154-1)
Supplement: Supplementary file 9 — Reporting Summary [file 41467_2018_8154_MOESM9_ESM.pdf]

nature research

Corresponding author(s): Roger J. Daly

Last updated by author(s): Dec 12, 2018

Reporting Summary

Nature Research wishes to improve the reproducibility of the work that we publish. This form provides structure for consistency and transparency in reporting. For further information on Nature Research policies, see Authors & Referees and the Editorial Policy Checklist.

Statistics

For all statistical analyses, confirm that the following items are present in the figure legend, table legend, main text, or Methods section.

n/a	Confirmed	
☐	☒	The exact sample size (*n*) for each experimental group/condition, given as a discrete number and unit of measurement
☐	☒	A statement on whether measurements were taken from distinct samples or whether the same sample was measured repeatedly
☐	☒	The statistical test(s) used AND whether they are one- or two-sided<br>*Only common tests should be described solely by name; describe more complex techniques in the Methods section.*
☒	☐	A description of all covariates tested
☐	☒	A description of any assumptions or corrections, such as tests of normality and adjustment for multiple comparisons
☐	☒	A full description of the statistical parameters including central tendency (e.g. means) or other basic estimates (e.g. regression coefficient) AND variation (e.g. standard deviation) or associated estimates of uncertainty (e.g. confidence intervals)
☐	☒	For null hypothesis testing, the test statistic (e.g. *F*, *t*, *r*) with confidence intervals, effect sizes, degrees of freedom and *P* value noted<br>*Give P values as exact values whenever suitable.*
☒	☐	For Bayesian analysis, information on the choice of priors and Markov chain Monte Carlo settings
☒	☐	For hierarchical and complex designs, identification of the appropriate level for tests and full reporting of outcomes
☒	☐	Estimates of effect sizes (e.g. Cohen's *d*, Pearson's *r*), indicating how they were calculated

Our web collection on statistics for biologists contains articles on many of the points above.

Software and code

Policy information about availability of computer code

Data collection	XCalibur provided by Thermo was used to generate and collect MS data. Image Lab™ Touch Software (version 1.2.0.12) was used to collect western blot results. LAS AF (version 2.6.3) and ZereneStacker (version 1.04) were used to collect and stack the images of acini in 3D functional screen assay. LAS X (version 3.1.1.15751) was used to collect images in IF assay.
Data analysis	MaxQuant (version 1.2.2.5 and version 1.5.2.8) was used to analyse raw MS files. ImageJ (version 1.51) was used to measure acini size. Image Lab (version 5.2.1) and ImageJ (version 2.0.0) were used to analyse the densitometry of blots. Prism (version 7) was used to perform Mann-Whitney test and ANOVA assay. Prism (version 7) and Microsoft Excel (version 15.41) were used to perform Two-tailed Student's t-test.

For manuscripts utilizing custom algorithms or software that are central to the research but not yet described in published literature, software must be made available to editors/reviewers. We strongly encourage code deposition in a community repository (e.g. GitHub). See the Nature Research guidelines for submitting code & software for further information.

Data

Policy information about availability of data

All manuscripts must include a data availability statement. This statement should provide the following information, where applicable:

- Accession codes, unique identifiers, or web links for publicly available datasets
- A list of figures that have associated raw data
- A description of any restrictions on data availability

The mass spectrometry proteomics data have been deposited to the ProteomeXchange Consortium via the PRIDE partner repository with the dataset identifier PXD010687. All other data supporting the findings of this study are available from the corresponding author on reasonable request.

Field-specific reporting

Please select the one below that is the best fit for your research. If you are not sure, read the appropriate sections before making your selection.

☒ Life sciences ☐ Behavioural & social sciences ☐ Ecological, evolutionary & environmental sciences

For a reference copy of the document with all sections, see nature.com/documents/nr-reporting-summary-flat.pdf

Life sciences study design

All studies must disclose on these points even when the disclosure is negative.

Sample size	No sample-size calculation was performed. Mice numbers were determined by experience in Xenografts study.
Data exclusions	No data were excluded.
Replication	All attempt at replication were successful.
Randomization	Mice came as 8 per cage and 8 mice in each cage were used as a experimental group.
Blinding	To give different treatments to different experimental groups, the investigators were not blinded in Xenografts study.

Reporting for specific materials, systems and methods

We require information from authors about some types of materials, experimental systems and methods used in many studies. Here, indicate whether each material, system or method listed is relevant to your study. If you are not sure if a list item applies to your research, read the appropriate section before selecting a response.

Materials & experimental systems

n/a	Involved in the study	
☐	☒	Antibodies
☐	☒	Eukaryotic cell lines
☒	☐	Palaeontology
☐	☒	Animals and other organisms
☒	☐	Human research participants
☒	☐	Clinical data

Methods

n/a	Involved in the study	
☒	☐	ChIP-seq
☒	☐	Flow cytometry
☒	☐	MRI-based neuroimaging

Antibodies

Antibodies used

Antibodies against beta-actin (1:5,000, catalog no. sc-69879), Pan 14-3-3 (1:10,000, catalog no. sc-1657) and MAP4K5 (KHS, 1:500, catalog no. sc-6429) were purchased from Santa Cruz Biotechnology (Dallas, TX). a-tubulin (1:5,000, catalog no. T5168) was purchased from Sigma. SGK1 (1:1,000, catalog no. 3272 and 12103), pErk-T202/Y204 (1:2,000, catalog no. 4370S), Erk (1:2,000, catlog no. 4695S), pJNK-T183/Y185 (1:1,000, catalog no. 9252), JNK (1:2,000, catalog no. 9251), p-p38-T180/Y182 (1:1,000, catalog no. 4511), p38 (1:2,000, catalog no. 9212), pYAP-S127 (1:2,000, catalog no. 4911), YAP (1:2,000, catalog no. 14074), pNDRG1-T346 (1:2,000, catalog no. 5482), NDRG1 (1:2,000, catalog no. 9485) pS6-S235/236 (1:1,000, catalog no. 2211), S6 (1:1,000, catalog no. 2217), pGSK3a/b-S21/9 (1:1,000, catalog no. 9331), pSrc-Y416 (1:1,000, catalog no. 2123) and Cleaved Caspase-3 (Asp175) (1:100 for IF, catalog no. 9664) were purchased from Cell Signaling Technology (Danvers, MA). Ki67 (1:200 for IF, catalog no. RM-9106) was purchased from Thermo Fisher. Donkey anti-rabbit Alexa Fluor® 488 (1:500, catalog no. A-21206) was purchased from Life Technologies.

Validation

All antibodies have been validated by manufacturers and optimized under lab conditions on applications in this study. Details are as below.
beta-actin (1:5,000, catalog no. sc-69879), cited by >400 papers.
Pan 14-3-3 (1:10,000, catalog no. sc-1657), cited by >120 papers.
MAP4K5 (KHS, 1:500, catalog no. sc-6429), cited in Mol Cell Biol. 2006 Sep;26(17):6511-21 and Oncotarget. 2015 Oct 27;6(33):34629-48 with WB on human species samples. Validated for WB by our Lab by knocking down with siRNAs.
a-tubulin (1:5,000, catalog no. T5168), cited by >1000 papers.
SGK1 (1:1,000, catalog no. 3272), cited by 14 papers. Applied on WB on human species samples including but not limited to papers of Oncogene (2011) 30, 3198–3206; J Immunol. 2014 February 15; 192(4): 1796–1805 and Cell Physiol Biochem 2016;39:1295-1306. Validated for WB by our Lab by knocking down with siRNAs.
SGK1 (1:1,000, catalog no. 12103), cited by 6 papers. Applied on WB on human species samples including but not limited to papers of Nat Commun. 2016 Jan 5;7:10174; Oncotarget. 2017 Jan 24;8(4):5992-6002 and Oncotarget. 2017 Jul 11;8(28):46121-46135. Validated for WB by our Lab by knocking down with siRNAs.
pErk-T202/Y204 (1:2,000, catalog no. 4370S), cited by >2000 papers.
Erk (1:2,000, catlog no. 4695S), cited by >1500 papers.

pJNK-T183/Y185 (1:1,000, catalog no. 9252), cited by >1000 papers.
JNK (1:2,000, catalog no. 9251), cited by >1000 papers.
p-p38-T180/Y182 (1:1,000, catalog no. 4511), cited by >600 papers.
p38 (1:2,000, catalog no. 9212), cited by >1500 papers.
pYAP-S127 (1:2,000, catalog no. 4911), cited by >180 papers.
YAP (1:2,000, catalog no. 14074), cited by >55 papers.
pNDRG1-T346 (1:2,000, catalog no. 5482), cited by >30 papers.
NDRG1 (1:2,000, catalog no. 9485), cited by 11 papers. Applied on WB on human species samples including but not limited to papers of Nat Commun. 2016 Jan 5;7:10174; Breast Cancer Res. 2018 Jun 14;20(1):55B; Sci Rep. 2017; 7: 44602 and Cell Death Dis. 2017 Sep; 8(9): e3048.
pS6-S235/236 (1:1,000, catalog no. 2211), cited by >500 papers.
S6 (1:1,000, catalog no. 2217), cited by >500 papers.
pGSK3a/b-S21/9 (1:1,000, catalog no. 9331), cited by >270 papers.
pSrc-Y416 (1:1,000, catalog no. 2123), cited by >70 papers.
Cleaved Caspase-3 (Asp175) (1:100 for IF, catalog no. 9664), cited by >1300 papers.
Ki67 (1:200 for IF, catalog no. RM-9106) was purchased from Thermo Fisher. Applied on IF or IHC on human species samples including but not limited to papers of Nat Commun. 2015 Jan 23;6:6001; Nature. 2017 Jan 12;541(7636):228-232 and Oncogene. 2013 Jan 17; 32(3): 286–295.

Eukaryotic cell lines

Policy information about cell lines

Cell line source(s)	MCF-10A EcoR cells were obtained from Brugge lab (Harvard) and described in R. J. Daly, 2006; JBC, 281, 626-637. A549 cells were obtained from Professor Jian Li lab (Monash Biomedicine Discovery Institute) and described in J. Li, 2017; Antimicrobial Agents and Chemotherapy, 61, e02690-16. All other breast cancer cell lines were obtained from the American Type Culture Collection, except for MDA-MB-231 (EG&G Mason Research Institute, Worchester, MA) and described in R. J. Daly, 2013; Cancer Res, 73, 1969-1980.
Authentication	Cell lines were authenticated by short tandem repeat polymorphism, single-nucleotide polymorphism, and fingerprint analyses.
Mycoplasma contamination	All cell lines were negative in mycoplasma testing.
Commonly misidentified lines (See ICLAC register)	No commonly misidentified lines used.

Animals and other organisms

Policy information about studies involving animals; ARRIVE guidelines recommended for reporting animal research

Laboratory animals	4-6 weeks female BALB/C nude mice were used in this project.
Wild animals	The study did not involve wild animals.
Field-collected samples	The study did not involve samples collected from the field.
Ethics oversight	All procedures involving mice were conducted in accordance with National Health and Medical Research Council (NHMRC) regulations on the use and care of experimental animals and the study protocol approved by the Monash University Animal Ethics Committee.

Note that full information on the approval of the study protocol must also be provided in the manuscript.
